# CD8-positive lymphocyte infiltration as a marker of anti-tumor immune response in rectal cancer: pre- and post-neoadjuvant radiotherapy comparison

**DOI:** 10.1016/j.ctro.2025.101018

**Published:** 2025-07-16

**Authors:** Samaneh Salarvand, Romina Abyaneh, Abdorreza Raee, Mahdieh Yaghooti-Khorasani, Fariba Mohammadjani, Fatemeh Nili, Mahdi Aghili, Reza Ghalehtaki

**Affiliations:** a Department of Anatomical and Clinical Pathology, IKHC, Tehran University of Medical Sciences Tehran, Iran; b Radiation Oncology Research Center, Cancer Research Institute, IKHC, Tehran University of Medical Sciences Tehran, Iran; c Department of Radiation Oncology, Cancer Institute, IKHC, Tehran University of Medical Sciences Tehran, Iran

**Keywords:** Rectal cancer, Neoadjuvant radiotherapy, Tumor-infiltrating CD8-positive lymphocytes

## Abstract

**Background:**

Antitumor immunity, exerted by CD8+ cytotoxic T lymphocytes, plays a vital role in tumor control. Therefore, the present study was conducted to compare the amount of CD8+ tumor-infiltrating lymphocytes (TILs) before and after either long- (LCRT) or short-course radiotherapy (SCRT) in rectal cancer.

**Methods:**

This study retrospectively assessed rectal cancer patients treated by neoadjuvant radiotherapy between 2019 and 2021. Biopsy and surgical samples were subjected to immunohistochemical staining to count CD8+ TILs. The association between the post-to-pre-treatment CD8+ count ratio and treatment groups, histopathological factors, and response to treatment was assessed.

**Results:**

A total of 34 patients were included, with 23 (67.6 %) receiving LCRT and 11 (32.4 %) receiving SCRT. The mean age was 58.56 ± 13.59 years. The number and percentage of CD8+ TILs increased significantly after radiotherapy in all patients (P < 0.001). An increase in CD8+ TILs was observed in both groups, with LCRT showing a median post-to-pre-treatment count ratio of 2.77 and SCRT showing 3.1 (P = 0.127). A generalized linear multivariate model adjusting for mucinous histology, surgical grade, and pathological stages revealed that SCRT was associated with a significantly higher post-to-pre-treatment CD8+ count ratio compared to LCRT (P = 0.03).

**Conclusion:**

Our study highlights that both SCRT and LCRT significantly increase CD8+ TIL count and percentage, reflecting robust immune activation after radiotherapy in rectal cancer, with SCRT showing a higher relative increase, though not statistically significant in unadjusted analyses. After adjusting for histopathological variables, SCRT was independently associated with a greater increase in CD8+ T cells.

## Highlights

• Neoadjuvant radiotherapy significantly increased CD8+ TILs.
• SCRT showed a significantly higher CD8+ TIL fold-change after adjustment for confounders.
• Histopathologic factors were not associated with CD8+ TIL dynamics.

## Introduction

Rectal cancer was historically treated with surgery alone, but management has evolved toward multimodal approaches. For locally advanced rectal cancer (LARC), neoadjuvant chemoradiotherapy followed by surgery has become the standard of care, and total neoadjuvant therapy (TNT) is now increasingly being adopted to improve outcomes [[1], [2], [3], [4], [5], [6]]. Radiotherapy can be administered in either a short-course or long-course schedule. Short-course radiotherapy (SCRT) delivers a total dose of 25 Gy, given as 5 Gy per day over 5 days, followed by an early or delayed surgery. In contrast, long-course radiotherapy (LCRT) involves a total dose of 45–50.4 Gy, delivered daily over 5–6 weeks, with surgery performed at least 4–6 weeks after the completion of radiotherapy [7].

Chemotherapy and radiotherapy were previously perceived to have immunosuppressive effects due to their impact on lymphocytes. However, recent research suggests that these treatments can augment systemic anti-tumor immune effects by triggering immunogenic cell death and promoting T-cell responses [8,9]. Tumor-infiltrating lymphocytes (TILs) are commonly found in the tumor tissue and surrounding cells, as part of the host's primary immune response to malignant colorectal tumors [10,11]. The presence of CD8+ cytotoxic T lymphocytes is crucial for suppressing cancer development and controlling disease progression [12], leading to improved survival in various cancers, including melanoma, non-small cell lung cancer, urothelial cancer, as well as gastrointestinal cancers [[13], [14], [15], [16], [17], [18], [19]].

A number of studies have also shown a correlation between significant lymphocytic infiltration and a high response rate to radiotherapy and chemotherapy [20,21]. Also, Galon and colleagues introduced an immune scoring system as a prognostic criterion by evaluating the density of TILs within the tumor and reported that this system provides a more valuable prognostic criterion than conventional histopathological scoring, such as the TNM classification [22]. Also, the tumor immune microenvironment, particularly the presence and activity of CD8+ TILs, plays a critical role in determining immunotherapy outcomes, since cytotoxic T cells are the primary effectors of immune checkpoint inhibitors (ICIs) [23]. The majority of colorectal cancer cases have mismatch repair proficient (pMMR) or microsatellite stable (MSS) tumors [24], which are considered less sensitive to immunotherapy than mismatch repair-deficient (dMMR) cases, likely due to a lack of pre-existing CD8+ TILs. However, higher CD8+ PD-1+ T-cell infiltration predicted better response to ICIs in pMMR tumors [25]. Thus, evidence suggests that neoadjuvant strategies that promote CD8+ T-cell infiltration, such as (chemo)radiotherapy, may sensitize pMMR tumors to immunotherapy, which is a topic of recent clinical trials [[26], [27], [28], [29]].

The differences in immune response following various neoadjuvant treatments for rectal cancer and their clinical value have not been extensively studied. It is hypothesized that SCRT, which delivers a higher dose, stimulates the immune system more effectively, thereby increasing the anti-tumor lymphocyte response more than LCRT. Thus, this study aims to compare the anti-tumor immune response by examining the number of CD8+ TILs in tumor biopsy samples before and after neoadjuvant radiotherapy, using both SCRT and LCRT methods.

## Methods

### Patient characteristics and treatment protocol

This cross-sectional study aimed to retrospectively compare the anti-tumor immune response based on the CD8+ TIL count in the tissue samples of patients with rectal carcinoma prior to and following neoadjuvant radiotherapy, using either SCRT or LCRT. Patients diagnosed with rectal carcinoma who underwent neoadjuvant radiotherapy at the Cancer Institute of Iran between 2019 and 2022 were identified. The required demographic information, including the age and sex of the patients, was extracted from the clinical records of the patients in the pathology department. Based on their radiotherapy protocol, patients were categorized into two groups. The SCRT group received 25 Gy over five consecutive days, with concurrent 5-FU-based chemotherapy using capecitabine at a dose of 825 mg/m^2^ or CAPOX (capecitabine 625 mg/m^2^ BD + oxaliplatin 50 mg/m^2^), followed by surgery 8–12 weeks later. The LCRT group received a total dose of 45–50.4 Gy, delivered as 1.8 Gy per fraction over 5–6 weeks with concurrent chemotherapy by oral capecitabine (825 mg/m^2^ BD), followed by surgery after 8–12 weeks. Before or after chemoradiation, some patients received induction or consolidation chemotherapy. When capecitabine was used alone as part of these regimens, it was administered orally at 1000 mg/m^2^ twice daily from day 1 to day 14, every three weeks.

### Histopathological evaluation and immunohistochemistry

**Fig. 1:**
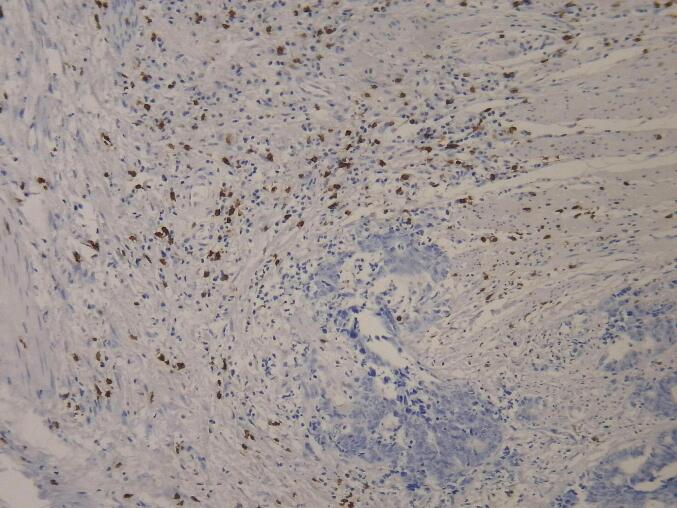
CD8-positive cells in surgical specimen, x200.

**Fig. 2:**
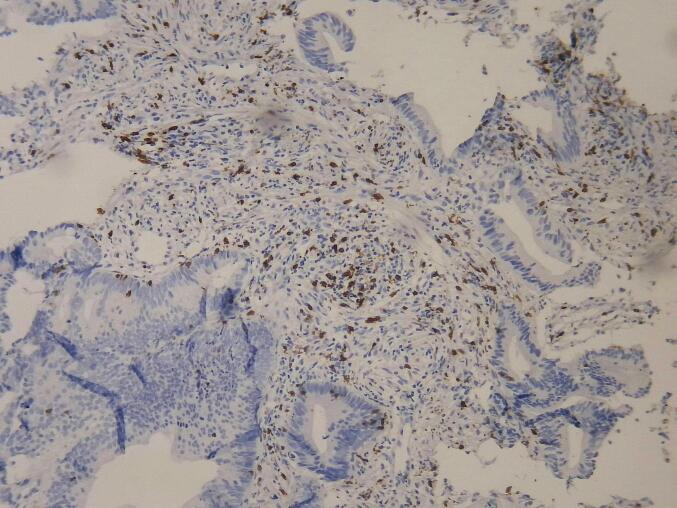
CD8-positive cells in biopsy specimen, x200.

Paraffin blocks and hematoxylin and eosin (H&E)-stained slides from pre- and post-neoadjuvant treatment samples were retrieved from the pathology department's archives and reviewed by a pathologist. A suitable paraffin block was selected for immunohistochemical (IHC) staining if minimal necrosis, minimal hemorrhage, and sufficient tumor tissue were present. Samples lacking an appropriate block for staining were excluded from the study. Fig. 1, Fig. 2 show the microscopic view of slides stained with CD8 antibody in biopsy and surgical samples.

Also, patients' histopathological information, including light microscope findings such as the pathological response to treatment, tumor regression grade (TRG), perineural invasion (PNI) status, lymphovascular invasion (LVI) status, the presence of a mucinous component, lymph node involvement, tumor size, and surgical margin involvement, were extracted from patients’ pathology reports. The post-treatment sample slides stained with the H&E method were confirmed by a light microscope. Also, the number of CD8 + TILs in the post- and pre-treatment samples was assessed using IHC and observed with a light microscope.

### Endpoints

The primary endpoint of this study was to evaluate the change in CD8+ TILs before and after neoadjuvant radiotherapy in patients with LARC. This was assessed both as absolute cell counts and as the percentage of CD8+ lymphocytes relative to total infiltrating cells.

The secondary endpoints included (1) comparing treatment response between the SCRT and LCRT groups, as measured by TRG, post-treatment T stage (ypT), and N stage (ypN); (2) examining the association between CD8+ TIL infiltration (pre- and post-treatment counts, and post-to-pre-treatment ratios) and various clinicopathological variables such as PNI, LVI, mucinous histology, surgical grade, and pathologic staging, TRG; and (3) identifying independent predictors of CD8 + TIL dynamics using multivariable modeling approaches.

### Statistical analysis

Statistical analysis was conducted using SPSS software version 27. Continuous variables were reported as mean ± standard deviation (SD) and compared using independent t-tests. Categorical variables were summarized as frequencies and percentages and compared using Pearson's chi-square or Fisher's exact tests, as appropriate. Pre-treatment CD8 + counts, which were normally distributed, were reported as mean ± SD and compared between the SCRT and LCRT groups using independent t-tests. Post-treatment CD8+ counts, post-treatment CD8+ percentages, pre-treatment CD8+ percentages, post/pre-treatment count ratios, and post/pre-treatment percentage ratios were non-normally distributed; thus, they were reported as median [interquartile range (IQR)] and compared between groups using the Mann-Whitney *U* test. Pre-to-post treatment comparisons within the whole population and within each treatment group were performed using Wilcoxon signed-rank tests. Statistical analyses of CD8+ T-cell infiltration patterns were conducted according to data distribution. Pre-treatment CD8+ counts were analyzed using independent t-tests for binary variables and one-way ANOVA for multi-category variables. Post-treatment CD8+ counts and post-to-pre-treatment ratios were analyzed using Mann-Whitney U tests and Kruskal-Wallis tests. All statistical tests were two-tailed, with significance set at p < 0.05. Additionally, variables with a p-value < 0.2 in univariate analyses were further evaluated using a generalized linear model to identify independent post-to-pre-treatment CD8+ count ratio predictors.

### Ethical considerations

This study adhered to all principles outlined in the Helsinki Declaration. The institutional review board and ethics committee have approved the study design (IR.TUMS.IKHC.REC.1399.471). Informed consent was obtained from all participants at the time of admission, allowing for the potential use of their information in research. The paraffin blocks and sample slides used in this study were previously employed for diagnostic purposes, thereby mitigating ethical concerns. No additional costs were incurred by patients for the tests conducted. The data contained in patient files was accessible only to the researcher, and to protect patient confidentiality, each case was assigned a specific code for identification.

## Results

### **Characteristics of patients and** treatments

A total of 34 patients were included; 11 (32.4 %) were female, and the mean age was 58.56 ± 13.59. A total of 23 (67.6 %) underwent LCRT, and 11 (32.4 %) underwent SCRT. In the LCRT group, all patients received concurrent capecitabine with radiotherapy. In the SCRT group, the concurrent chemotherapy regimen was unknown for one patient, while three patients did not receive any concurrent chemotherapy. Two patients in this group received CAPOX, and five received capecitabine as their concurrent chemotherapy agent. In the LCRT group, five patients underwent consolidation chemotherapy with CAPOX: three received one cycle, one received two cycles, and one received three cycles. In the SCRT group, nine patients received consolidation chemotherapy. Of these, six were treated with CAPOX (two received one cycle, one received three cycles, and the remaining three received four, five, and six cycles, respectively). One patient in the SCRT group received five cycles of FOLFOX, and another received three cycles of 5-FU plus leucovorin. Additionally, one patient in this group received two cycles of capecitabine as induction chemotherapy, followed by four cycles of capecitabine as consolidation therapy. Demographic and baseline histopathological findings of the patients in the two radiotherapy groups are presented in Table 1. There were no significant differences in the clinical and histopathological characteristics between the two groups.

**Table 1:** Demographic, Histopathologic, and Staging Comparison Between the Two Groups.

Variables	Results	Total	SCRT	LCRT	P value
**Mean age ± SD**	**Years**	58.56 ± 13.59	61.64 ± 12.37	57.09 ± 14.17	0.370
**RT to surgery interval (Median [IQR])**	**Weeks**	18 [9.5–26]	24 [11–29]	17 [8.75–23.5]	0.178
**Sex** **N (%)**	**Male** **Female**	23 (67.6 %)	6 (54.5 %)	17 (73.9 %)	0.434
		11 (32.4 %)	5 (45.5 %)	6 (26.1 %)	
**PNI** **N (%)**	**Positive** **Negative**	10 (29.4)	3 (27.3 %)	7 (30.4 %)	0.850
		24 (70.6 %)	8 (72.7 %)	16 (69.6 %)	
**LVI** **N (%)**	**Positive** **Negative**	13 (38.2 %)	5 (45.5 %)	8 (34.8 %)	0.549
		21 (61.8 %)	6 (54.5 %)	15 (65.2 %)	
**Mucinous** **N (%)**	**Positive** **Negative**	5 (14.7 %)	2 (18.2 %)	3 (13 %)	0.692
		29 (85.3 %)	9 (81.8 %)	20 (87 %)	
**Grade (n = 27)** **N (%)**	**1** **2** **3**	9 (29.2 %)	20	7 (30.4 %)	0.202
		15 (58.3 %)	7 (100 %)	8 (34.8 %)	
		3 (12.5 %)	0	3 (13 %)	
**Stage** **N (%)**	**T2** **T3** **T4**	3 (8.8 %)	1(9.1 %)	2 (8.7 %)	0.857
		29 (85.3 %)	9 (81.8 %)	20 (87 %)	
		2 (5.9 %)	1 (9.1 %)	1 (4.3 %)	
**LN involvement N (%)**	**Positive** **Negative**	26 (76.5 %)	9 (81.8 %)	17 (73.9 %)	0.611
		8 (23.5 %)	2 (18.2 %)	6 (26.1 %)	
**Mean tumor size ± SD**	**Centimeter**	3.16 ± 2	3.17 ± 1.84	3.26 ± 2.11	0.907
Abbreviations: RT: radiotherapy; PNI: perineural invasion; LVI: lymphovascular invasion; LN: lymph node. Data were present as					

### Treatment outcomes

Table 2 outlines the neoadjuvant treatment response in the entire cohort as well as the LCRT and SCRT groups, assessed through post-treatment pathological staging (ypT, ypN) and TRG. The results indicate no significant statistical differences between the two treatment groups in tumor response and pathological stages.

**Table 2:** Comparative Analysis of Treatment Response Between Groups.

variable	Results	Total	LCRT	SCRT	P value
**yPT** **N (%)**	**T0**	4 (11.8 %)	3 (13 %)	1 (9.1 %)	0.136
	**T1**	2 (55.9 %)	2 (8.7 %)	0	
	**T2**	6 (17.6 %)	2 (8.7 %)	4 (36.4 %)	
	**T3**	19 (55.9 %)	15 (65.2 %)	4 (36.4 %)	
	**T4**	3 (8.8 %)	1 (4.3 %)	2 (18.2 %)	
**yPN** **N (%)**	**N0**	22 (64.7 %)	16 (69.6 %)	6 (54.5 %)	0.142
	**N1**	9 (26.5 %)	4 (17.4 %)	5 (45.5 %)	
	**N2**	3 (8.8 %)	3 (13 %)	0	
**TRG** **N (%)**	**0**	5 (15.2 %)	4 (17.4 %)	1 (10 %)	0.097
	**1**	4 (12.1 %)	2 (8.7 %)	2 (20 %)	
	**2**	18 (54.5 %)	15 (65.2 %)	3 (30 %)	
	**3**	6 (18.2 %)	2 (8.7 %)	4 (40 %)	
Abbreviation: TRG: tumor regression grade; yPT: post-neoadjuvant T-stage; yPN: post-neoadjuvant N-stage.					

**Fig. 3:**
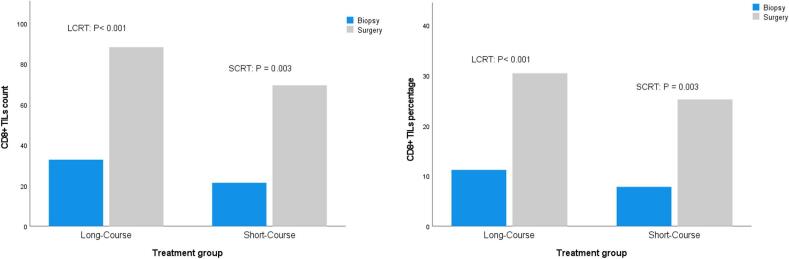
The number and percentage of CD8+ TILs before and after radiotherapy in the two groups were compared using a Wilcoxon Signed Ranks Test.

Table 3 presents the average number and percentage of CD8+ TILs before and after neoadjuvant treatment in the total cohort as well as SCRT and LCRT groups. All 34 examined samples showed a significant increase in the number and percentage of CD8+ lymphocytes after radiotherapy (Wilcoxon Signed Ranks Test, P < 0.001). Additionally, within each treatment group, the number and percentage of CD8+ lymphocytes relative to all infiltrating cells have significantly increased after radiotherapy (Wilcoxon Signed Ranks Test, SCRT: P = 0.003, LCRT: P < 0.001, Fig. 3). There was a significant difference in the number of CD8+ lymphocytes in pre-treatment samples (LCRT: 32.78 vs. SCRT: 21.36, P = 0.025), favoring higher numbers in the LCRT group, as well as a nonsignificant trend towards higher pre-treatment median CD8+ lymphocyte percentage in LCRT (10 % vs 6 %, P = 0.096); however, there were no statistically significant differences between the two treatment groups in the number or percentage of CD8+ lymphocytes in post-treatment samples. The LCRT and SCRT groups demonstrated a median post-to-pre-treatment count ratio of 2.77 and 3.1, respectively (P = 0.127). In terms of post-to-pre-treatment percentage ratio, the LCRT and SCRT groups showed a median post-to-pre-treatment percentage ratio of 2.70 and 3.60 respectively (P = 0.348).

**Table 3:** Dynamic Changes in CD8+ TILs: Counts, Percentages, and Post/Pre Ratios by Group.

CD8+ lymphocytes		Number before	Number after	P-value	Post/pre CD8+ TILs count ratio	Percentage before	Percentage after	P-value	Post/pre CD8+ TILs percentage ratio
Total	**Mean ± SD**	29.09 ± 14.081	82.18 ± 37.67	**<0.001**	4.06 ± 4.64	10.09 ± 5.48	28.71 ± 13.48	**<0.001**	4.63 ± 7.24
	**Median [IQR]**	30 [20–36.25]	76.5 [56.75–95.25]		2.93 [2.2–4.27]	10 [6–13]	27 [18.75–37.25]		2.77 [1.84–4.46]
	**Interval**	2–61	29–180		1.22–23.34	1–23	6–63		1.25–42
LCRT	**Mean ± SD**	32.78 ± 13.12	88.26 ± 41.89	**<0.001**	2.86 ± 1.18	11.17 ± 5.09	30.39 ± 14.01	**<0.001**	3.06 ± 1.71
	**Median [IQR]**	31 [26–37]	83 [60–107]		2.77 [1.97–4]	10 [7–13]	30 [20–38]		2.70 [1.18–4.33]
	**Interval**	13–61	29–180		1.22–5	3–23	6–63		1.25–8
SCRT	**Mean ± SD**	21.36 ± 13.34	69.45 ± 23.46	**0.003**	6.57 ± 7.61	7.82 ± 5.81	25.18 ± 12.14	**0.003**	7.92 ± 12.22
	**Median [IQR]**	22 [11–36]	67 [56–83]		3.1 [2.31–5.58]	6 [2–13]	26 [14–35]		3.6 [2–5.2]
	**Interval**	2–40	36–120		1.43–23.34	1–18	8–45		1.30–42
P value		**0.025**	0.224		0.127	0.096	0.299		0.348

The association between pre- or post-CD8+ counts and post-to-pre-treatment CD8+ count ratio and various histopathological factors is detailed in Table 4. Across all factors, including PNI, LVI, mucinous histology, surgical tumor grade, yPT, and yPN stages, no statistically significant differences were observed in pre- or post-treatment CD8+ TIL counts and post-to-pre-treatment CD8+ count ratio.

**Table 4:** Histopathological Associations with CD8+ TIL Infiltration Dynamics.

Variable	Results	CD8+ TILs before Mean ± SD	P value	CD8+ TILs after Median [IQR]	P value	Post/Pre CD8+ TILs count Ratio Median [IQR]	P value
PNI	Positive	30.40 ± 16.17	0.732	67.5 [58.75–85.25]	0.384	2.56 [2.19–3.14]	0.290
	Negative	28.54 ± 13.45		75 [46.25–101.75]		3.07 [2.40–4.85]	
LVI	Positive	27.54 ± 18.68	0.665	65 [47.50–85.50]	0.196	2.85 [2.15–3.75]	0.956
	Negative	30.05 ± 10.73		80 [60.5–98.50]		3 [2.32–3.90]	
Mucinous	Positive	27.60 ± 8.96	0.802	59 [31.25–82.25]	0.084	2.50 [1.43–3.07]	0.093
	Negative	29.34 ± 14.9		70 [59–95.25]		2.93 [2.33–4.27]	
Surgical grade	1	27.56 ± 12.36	0.974	75 [63.25–158.25]	0.318	3.65 [2.51–4.85]	0.159
	2	28.33 ± 16.33		67 [43–85]		2.85 [2.28–3.27	
	3	26.33 ± 9.86		65 [29-NA]		1.97 [1.93-NA]	
yPT	T0	32.25 ± 19.25	0.411	101.50 [75.50–120]	0.588	4.44 [1.91–5.31]	0.168
	T1	43.50 ± 9.19		99.50 [45-NA]		2.15 [1.21-NA]	
	T2	29.50 ± 11.43		70 [51–177]		3.17 [2.18–4.55]	
	T3	28.47 ± 13.91		68.50 [52–85.25]		2.45 [2–3.1]	
	T4	18.33 ± 14.18		70 [65-NA]		4.51 [3.1-NA]	
yPN	N0	29.82 ± 13.77	0.660	71.50 [59.25–108.75]	0.594	2.93 [1.96–4.43]	0.167
	N1	25.78 ± 14.78		81 [46.25–85.25]		3.09 [2.85–4.48]	
	N2	33.67 ± 17.80		43 [38-NA]		2.02 [1.26-NA]	
TRG	0	32 ± 16.68	0.610	120 [78–130]	0.124	4.51 [2.61–5.17]	0.530
	1	35 ± 5.6		70 [48–155.75]		2.25 [1.27–4.51]	
	2	28.39 ± 12.19		75 [58.75–95.25]		2.81 [2.27–3.30]	
	3	23.33 ± 21.62		60.50 [39.50–73.75]		2.76 [1.36–20.83]	
Abbreviations: PNI: perineural invasion; LVI: lymphovascular invasion; LN: lymph node; yPT: post neoadjuvant T-stage; yPN: post neoadjuvant N. TRG; tumor regression grade. Data were presented as mean ± SD for normally distributed and median [IQR] for not normally distributed variables.							

### Independent predictors of post-to-pre-treatment CD8 lymphocyte dynamics

To examine the association between the post-to-pre-treatment CD8+ count ratio and other histopathological variables, the Kolmogorov-Smirnov test was conducted, revealing that the independent quantitative variables, post-to-pre-treatment CD8+ count ratio, is not normally distributed.

In the univariate analysis of the entire population, the post-to-pre-treatment CD8+ count ratio was evaluated concerning various surgical and baseline histopathological variables. No significant associations were observed between the post-to-pre-treatment CD8+ count ratio and variables such as sex, radiotherapy approach (SCRT vs. LCRT), LVI status, PNI status, mucinous versus non-mucinous tumor histology, clinical node status, clinical T and N stages, ypT stage, ypN stage, TRG, or surgical grade.

However, a generalized linear model was utilized for those variables with a p-value less than 0.2 in the univariate analysis (including RT type, mucinous histological status, ypT stage, ypN stage, surgical grade) to further assess their potential as independent predictive markers of immune response. A Gamma generalized linear model (log link) adjusted for mucinous histology, surgical grade, and pathological stages revealed that LCRT was associated with significantly lower post-to-pre-treatment CD8+ count ratio compared to SCRT (β = -0.78, 95 % CI: −1.50 to −0.07; P = 0.03). No other variables reached statistical significance. The model demonstrated good fit (deviance/df = 0.43) and explained a significant proportion of the variance (P = 0.007). These findings suggest that SCRT may better preserve intratumoral CD8+ T-cell infiltration, warranting further investigation into its immunomodulatory effects.

## Discussion

In this study, we investigated the impact of neoadjuvant radiotherapy, using either SCRT or LCRT, on the anti-tumor immune response in patients with rectal carcinoma by assessing CD8+ TILs. Our findings demonstrated that both SCRT and LCRT significantly increased the number and proportion of CD8+ TILs following neoadjuvant therapy. The post-treatment CD8+ TIL counts and the magnitude of CD8+ increase were comparable between the two approaches. However, after adjustment for potential confounders, the post-to-pre-treatment CD8+ TIL ratio was significantly higher in the SCRT group, suggesting that SCRT may better enhance intratumoral immune responses.

Our results align with previous studies that highlight the immunomodulatory effects of radiotherapy, particularly its ability to promote CD8+ T-cell infiltration within the tumor microenvironment [[30], [31], [32]]. Shinto et al. observed a near doubling of stromal CD8+ lymphocyte density after SCRT [33]. Wang et al., using CT26 mouse models and human samples, showed an increase in CD8+ and CD4+ TILs within the tumors. Meanwhile, circulating T-lymphocytes did not change significantly in human samples, although CD4+ and CD8+ TILs increased after SCRT, confirming its immunostimulatory effects [34]. However, it is important to note that most of these studies assessed the overall impact of radiotherapy without directly comparing SCRT and LCRT, which limits their ability to clarify potential differences in the immunomodulatory dynamics between the two approaches.

A study by Hillson et al. examined immune responses in LARC patients receiving either LCRT (45 Gy) with concurrent capecitabine or SCRT (25 Gy) followed by 5-FU/oxaliplatin chemotherapy (SCRT-C). Serial analysis of tumor tissue and blood revealed that LCRT led to a consistent decrease in both cytotoxic (CD8+) and regulatory (FOXP3+) T cells during treatment, with partial recovery by 12 weeks. In contrast, early increases in both CD8+ and FOXP3+ TILs were observed in patients receiving SCRT-C, along with less pronounced reductions in circulating lymphocytes and no significant changes in immunostimulatory cytokines. Similar to our findings, the Hillson study suggested that SCRT-C may preserve or even enhance local immune activity, potentially offering a more immunogenic profile than LCRT [35].

Lim et al. evaluated T-lymphocyte subsets in patients undergoing neoadjuvant radiotherapy, observing a trend toward increased CD8+ lymphocyte counts specifically after LCRT. However, no significant changes were noted after SCRT. Surgery in their study was performed relatively early, 1 week after SCRT and 4–6 weeks after LCRT completion—a period during which immune suppression may still be ongoing (7), as it is known that radiotherapy can both stimulate and suppress immune responses in the tumor microenvironment, depending on the involved cell types. Studies show that CD8+ T cells increase in surgical samples 6–8 weeks after neoadjuvant radiotherapy, highlighting their role in anti-tumor immunity. In contrast, immune suppression is noted in samples taken 3–4 weeks post-treatment [[36], [37], [38]]. In our cohort, delayed surgery potentially allowed more time for immune recovery and CD8+ TIL expansion. This difference in surgical timing may partly explain the more robust increase in CD8+ TILs observed in our study across both SCRT and LCRT groups. Furthermore, differences in sample size, patient selection, and methods of TIL quantification may also contribute to the observed variations.

In contrast to our study, a study by Mirjolet and colleagues showed no variation in CD8+ TILs after preoperative RT. However, a significant decrease in FoxP3+ TILs was observed for both intraepithelial and stromal FoxP3 + TILs, but with a lower infiltration intensity concerning intraepithelial FoxP3+ TILs. The CD8+/FoxP3+ TILs ratio was significantly increased by preoperative RT. This increase concerned stromal CD8+/FoxP3+ TILs but not intraepithelial CD8+/FoxP3+ TILs. Additionally, the quantities of CD8+ and FoxP3+ TILs did not differ significantly between the two approaches; however, the CD8+/FoxP3+ TILs ratio was significantly higher following LCRT than SCRT, particularly within the stromal tissue, suggesting a stronger immune response with the LCRT approach. Additionally, a significant decrease in the CD8+/FoxP3+ TILs ratio was associated with improved progression-free survival (PFS) and overall survival (OS). The study also noted that the delay between radiotherapy and surgery was longer for LCRT (44.5 days) than for SCRT (35.1 days). However, the authors found that this timing did not significantly influence TIL variations [39].

Additionally, a study by Chen et al. provides important evidence on how the selection of neoadjuvant regimens and the timing of surgery influence the tumor immune microenvironment in rectal cancer. Patients received one of four approaches: (1) SCRT with immediate surgery within 1 week; (2) SCRT followed by capecitabine-based consolidation chemotherapy and delayed surgery; (3) SCRT followed by consolidation mFOLFOX6 and delayed surgery; or (4) LCRT (with concurrent capecitabine or 5-FU) with delayed surgery (without consolidation chemotherapy). Notably, increased stromal CD3+  and CD8+ T-cell infiltration was observed in the three arms with delayed surgery, regardless of whether patients received SCRT or LCRT, compared to the immediate surgery group. Importantly, high post-treatment stromal CD8+ T-cell densities correlated with improved tumor regression, lower distant metastasis rates, and longer disease-free survival (DFS), suggesting that enhanced cytotoxic immune activation plays a key prognostic role across treatment strategies [40].

In our analysis, we found no significant difference in baseline CD8+ T-cell infiltration across a range of pathological variables, including TRG, ypT, and ypN. Although there was a numerically higher mean CD8+ count in patients achieving TRG 0–1 compared to TRG 2–3, the difference did not reach statistical significance. Similarly, CD8+ counts did not differ significantly across ypT or ypN stages, suggesting that reliance on a single biopsy sample for pre-treatment analysis presents a limitation, as the sampled area may not represent the overall tumor, given the variability in inflammatory cell density across different regions. However, previous studies offer differing perspectives. In a retrospective study by Yasuda and colleagues, the density of CD4+ and CD8+ TILs in biopsy samples before CRT was evaluated. Higher TIL densities were strongly associated with greater tumor reduction ratios and higher post-treatment histological grades, with CD8+ TIL density identified as an independent predictor of complete response [21]. Additionally, Teng et al. linked higher pre-treatment CD8+ TIL counts to better TRG, DFS, and OS [31]. Also, Matsutani et al. reported that low pre- and post-treatment CD8+ TIL densities were associated with poor response, suggesting prognostic relevance [32]. One possible explanation lies in the dynamic nature of the immune microenvironment, where CD8+ T-cell functionality, activation status, and spatial distribution may matter more than pure quantity [41,42]. Furthermore, differences in immune contexture across rectal tumors, treatment regimens may contribute to discrepancies in the literature.

Radiotherapy and chemotherapy boost systemic antitumor immunity by combining direct tumor cell killing with immune activation. They reduce immune suppression and promote the recruitment of cytotoxic CD8+ T cells, key players in tumor elimination [[43], [44], [45], [46]].

**Fig. 4:**
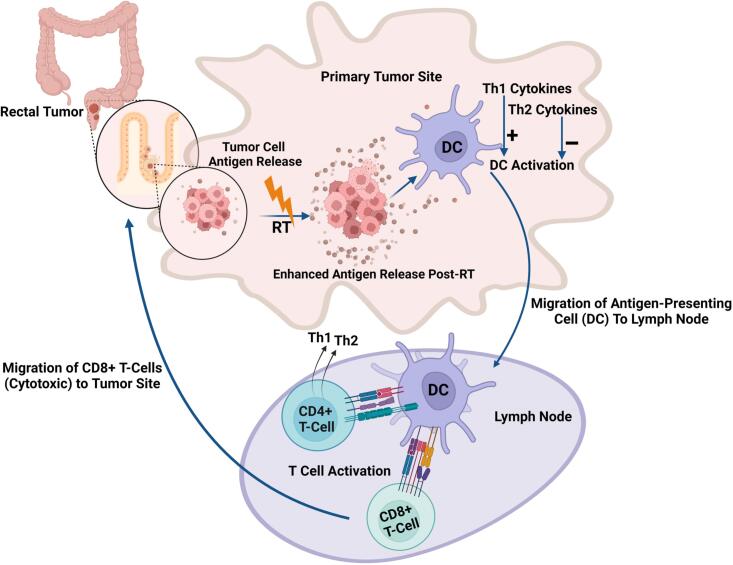
Anti-tumor immune regulation mechanism caused by radiotherapy Fig. 4**legend**: Untreated tumors express only a limited number of tumor-associated antigens. Neoadjuvant therapies can induce necrotic tumor cell death, which releases additional antigens and ultimately triggers an immune response. Radiotherapy facilitates this process by releasing tumor antigens, enhancing their presentation on the tumor cell surface, and attracting immune mediators, including pro-inflammatory cytokines such as interferon-gamma, which promote the activation of antigen-presenting cells (APCs) and further amplify the local immune response. T-helper 1 cytokines enhance this activation, while T-helper2 cytokines inhibit it. Once activated, APCs migrate to the draining lymph nodes, where they stimulate T-lymphocytes, leading to increased size and granularity of these cells. These now-activated, tumor-specific CD8+ cytotoxic T cells then return to the tumor microenvironment to effectively attack targeted tumor cells.

Fig. 4 depicts how radiotherapy stimulates antitumor immunity. Untreated tumors express only a limited number of tumor-associated antigens. By inducing necrotic tumor cell death, radiotherapy increases the level of tumor antigens released and pro-inflammatory cytokines, such as interferon-gamma, which activate antigen-presenting cells (APCs). APCs then migrate to lymph nodes to prime T cells, particularly CD8+ cytotoxic T cells, which return to the tumor to eliminate cancer cells. This response is enhanced by T-helper 1 cytokines and inhibited by T-helper 2 cytokines [47,48].

Since activated CD8+ lymphocytes can directly kill tumor cells and play an essential role in anti-tumor immunity, the combination of neoadjuvant chemotherapy and radiotherapy with immunotherapy may enhance the T cell-dependent antitumor response [49]. Modulation of signaling through stimulatory or inhibitory receptors on Τ-lymphocytes is considered a powerful method to influence anti-tumor immune responses. Ipilimumab, a cytotoxic T-lymphocyte-associated antigen-4 (CTLA-4) inhibitor, has been approved by the US Food and Drug Administration to treat unresectable or metastatic melanoma. Other agents that inhibit programmed death protein 1 (PD-1) and its ligand, programmed death ligand 1(PD-L1), are also in clinical development [50]. Also, primary results from a recent phase III randomized clinical trial evaluated the safety and pathological response of SCRT followed by total mesorectal excision (TME) and six cycles of adjuvant immunochemotherapy (CAPOX and camrelizumab) versus LCRT followed by TME and adjuvant chemotherapy. SCRT combined with immunochemotherapy significantly improved pCR rates compared to LCRT with adjuvant chemotherapy, while maintaining an acceptable safety profile in both treatment groups [51]. Additionally, long-term results from a single-arm phase II clinical trial demonstrated favorable survival rates after SCRT followed by the same chemoimmunotherapy regimen and delayed radical surgery [52].

A recent phase II randomized trial, the PRIME-RT trial, compared the response rates in patients with high-risk LARC treated with SCRT plus durvalumab, followed by FOLFOX and durvalumab, versus LCRT with capecitabine and durvalumab, followed by FOLFOX and durvalumab [53]. Total clinical/pathological complete response (CR: cCR/pCR) rate was 52 %, exceeding the predefined efficacy threshold, suggesting that TNT with immunotherapy is effective in patients with high-risk LARC. The SCRT arm showed numerically higher CR rates at 6 months and more durable responses at 1 year compared to LCRT, suggesting SCRT, combined with immunotherapy, might have a more favorable immunomodulatory effect and could be a promising strategy for organ preservation. (NCT04621370) [54].

A recent systematic review and meta-analysis highlighted a high level of CD8+ TILs as a significant predictor of improved survival in cancer patients undergoing ICI therapy. This association was consistent across various treatment strategies (immunotherapy alone, in combination, or as part of immunochemotherapy), cancer types such as lung, sarcoma, and melanoma, and different spatial distributions of CD8+ T cells within the tumor microenvironment, including intra-tumoral, stromal, or at the invasive margin. Notably, circulating CD8+ T cells in peripheral blood did not show this predictive value, emphasizing the importance of local immune activity within the tumor microenvironment for an effective ICI response [55]. While most evidence centers on solid tumors like lung and melanoma, these findings open up potential applications for rectal cancer treatment. Specifically, CD8+ TILs may serve as valuable biomarkers to predict response to combined radiotherapy and immunotherapy in rectal cancer, paving the way for more tailored therapeutic approaches.

This study has several strengths, including its paired design evaluating CD8+ TILs before and after neoadjuvant radiotherapy, comparison of SCRT and LCRT regimens, and adjustment for confounders using a multivariable model. Additionally, the study addressed the heterogeneity of infiltrating lymphocytes by averaging counts from five different fields. In cases with complete response to neoadjuvant therapy, lymphocytes were counted in the fibrotic and scarred areas caused by radiotherapy. However, no established method exists for counting inflammatory cells in such samples with a complete treatment response. On the other hand, the study is limited by a small sample size, a retrospective single-center design, and the lack of functional or spatial immune profiling as well as survival analysis.

## Conclusions

In this cohort of rectal cancer patients, both SCRT and LCRT approaches significantly increased the number and percentage of CD8+ T-cell infiltration after radiotherapy. Although in unadjusted analyses, SCRT was not associated with a higher increase in CD8+ T-cell infiltration compared to LCRT, after adjustment for key histopathological variables using a generalized linear model, SCRT was independently associated with a greater relative increase in CD8+ T-cell infiltration. These findings suggest that SCRT may exert a stronger immunomodulatory effect in favor of CD8+ TILs, potentially enhancing antitumor immune responses. Further investigation in larger cohorts is warranted to validate these results and explore their therapeutic relevance, particularly in the context of combining immunotherapy with chemoradiotherapy.

## Ethical Approval and Consent to Participate

All participants provided written informed consent at admission for the potential use of their data in research.

## CRediT authorship contribution statement

**Samaneh Salarvand:** Conceptualization, Investigation, Methodology, Validation, Resources, Writing – review & editing. **Romina Abyaneh:** Methodology, Resources, Data curation, Formal analysis, Writing – original draft, Visualization, Writing – review & editing. **Abdorreza Raee:** Investigation, Resources, Writing – original draft, Writing – review & editing. **Mahdieh Yaghooti-Khorasani:** Investigation, Writing – review & editing. **Fariba Mohammadjani:** Conceptualization, Investigation, Methodology, Validation, Resources, Writing – review & editing. **Fatemeh Nili:** Conceptualization, Investigation, Methodology, Validation, Resources, Writing – review & editing. **Mahdi Aghili:** Validation, Writing – review & editing. **Reza Ghalehtaki:** Conceptualization, Supervision, Project administration, Methodology, Validation, Resources, Formal analysis, Writing – review & editing.

## Funding

No funding was received to conduct this study.

## Declaration of competing interest

The authors declare that they have no known competing financial interests or personal relationships that could have appeared to influence the work reported in this paper.

## Data Availability

The datasets used or analyzed during the current study are available from the corresponding author upon reasonable request.
